# Profile of nursing graduates: competencies and professional insertion*

**DOI:** 10.1590/1518-8345.3222.3205

**Published:** 2019-10-28

**Authors:** Amanda Conrado Silva Barbosa, Franciane Silva Luiz, Denise Barbosa de Castro Friedrich, Vilanice Alves de Araújo Püschel, Beatriz Francisco Farah, Fábio da Costa Carbogim

**Affiliations:** 1 Instituto Federal de Educação, Ciência e Tecnologia do Sudeste de Minas Gerais, São João del-Rei, MG, Brazil.; 2 Universidade Federal de Juiz de Fora, Programa de Pós-Graduação em Enfermagem, Juiz de Fora, MG, Brazil.; 3 Universidade Federal de Juiz de Fora, Faculdade de Enfermagem, Juiz de Fora, MG, Brazil.; 4 Universidade de São Paulo, Escola de Enfermagem, São Paulo, SP, Brazil.

**Keywords:** Education, Nursing, Students, Nursing, Curriculum, Job Market, Health Manpower, Professional Competence, Educação em Enfermagem, Estudantes de Enfermagem, Currículo, Mercado de Trabalho, Recursos Humanos em Saúde, Competência Profissional, Educación en Enfermería, Estudiantes de Enfermería, Curriculum, Mercado de Trabajo, Recursos Humanos en Salud, Competencia Profesional

## Abstract

**Objective:**

evaluate the profile of the graduates of Nursing a public college from the perception of skills developed during graduation and the process of professional insertion.

**Method:**

quantitative, exploratory and descriptive study. The sample was composed of 216 graduates. The data was collected by a validated questionnaire and sent to a population of 470 egresses via electronic mail. For the analysis of the data, frequencies, mean and standard deviation were applied and, for the correlation, the chi-square test.

**Results:**

the majority of the participants were female (88%) and the mean age was 29.62 years. The majority (65%) had an employment relationship, 14% worked in a single institution and 48% started working six months after graduation. Regarding the form of work, 56% work in care, with an average of 4.5 minimum wages and a weekly workload between 37 and 44 hours. The majority reported competence acquisition to practice the profession, assisting the patient in his integrality with ethics and applying technical and scientific concepts in care.

**Conclusion:**

the study made it possible to describe the singularities of nurses’ education, their insertion in the world of work and the impact on the educational institution, as well as the presentation of specific competences from the perspective of the graduates themselves.

## Abstract (pt)

**Objetivo:**

avaliar o perfil dos egressos de Enfermagem uma faculdade pública a partir da percepção de competências desenvolvidas durante a graduação e do processo de inserção profissional.

**Método:**

estudo quantitativo, exploratório e descritivo. Compôs-se a amostra por 216 egressos. Os dados foram coletados por questionário validado e encaminhado a uma população de 470 egressos via correio eletrônico. Para a análise dos dados, aplicaram-se frequências, média e desvio-padrão e, para a correlação, o teste qui-quadrado.

**Resultados:**

a maioria dos participantes era do sexo feminino (88%) e a média de idade foi de 29,62 anos. A maioria (65%) possuía vínculo empregatício, 14% trabalham em uma única instituição e 48% começaram a trabalhar seis meses após a conclusão da graduação. Quanto à forma de trabalho, 56% atuam na assistência, com média de 4,5 salários mínimos e carga horária semanal entre 37 e 44 horas. A maioria reportou aquisição de competência para exercer a profissão, assistindo o paciente na sua integralidade com ética e aplicando conceitos técnicos e científicos no cuidado.

**Conclusão:**

o estudo viabilizou a descrição das singularidades da formação do enfermeiro, da inserção no mundo do trabalho e o impacto para a instituição educacional formadora, bem como a apresentação das competências específicas a partir da ótica dos próprios egressos.

## Abstract (es)

**Objetivo:**

evaluar el perfil de egresados del curso de enfermería de una universidad pública, a partir del análisis de las competencias adquiridas durante su formación académica, así como de su proceso de inserción profesional.

**Método:**

estudio cuantitativo, exploratorio y descriptivo. La muestra fue realizada con 216 egresados. Los datos se recolectaron por medio de cuestionario validado y encaminado vía correo electrónico a 470 egresados. Para el análisis de los datos se consideraron frecuencias, media y desviación estándar y para la correlación se aplicó la prueba chi-cuadrado.

**Resultados:**

la mayor parte de los participantes era de sexo femenino (88%) con un promedio de edad de 29,62 años. La mayoría (65%) contaba con vínculo laboral, un 14% trabajaba en una única institución y un 48% comenzó a trabajar seis meses después de la conclusión de los estudios universitarios. En relación a la forma de trabajo, el 56% actúa en la asistencia, con un ingreso promedio de 4,5 salarios mínimos y una carga horaria semanal de 37 a 44 horas. La mayoría reportó haber adquirido competencia para ejercer su profesión atendiendo a los pacientes de forma integral, ética y aplicando conceptos técnicos y científicos para su cuidado.

**Conclusión:**

el estudio posibilitó describir singularidades de la formación del enfermero, su inserción en el mundo laboral y el impacto para la institución educativa formadora, así como la presentación de competencias específicas desde la óptica de los propios egresados.

## Introduction

The training of health professionals, throughout Brazil’s historical trajectory, has been inclined to follow the demands of the world of work and the political, social and economic context. In this sense, the curricular organization of the training schools, with a view to attending to the dominant biomedical model, sometimes contributed to a technical, hospital and curative education ^(^ 1 ^)^. This model approaches the Flexnerian standard, created in the United States in 1910, to attend to the medical-industrial complex, based on the professionals’ precocious specialization, to act recovering mismatches^(^ 1 ^)^.

Nevertheless, with the movement of social medicine^(^ 2 ^-^ 3 ^)^ and the creation of the Unified Health System (UHS), training was redirected with a view to promoting, preventing and recovering health, with a focus on the community^(^ 3 ^-^ 4 ^)^. In addition, there was a stimulus in the curricula bases to the essential competences, like the critical thinking, the initiative, the autonomy, the creativity and the problem solving capacity^(^ 4 ^-^ 5 ^)^. Competencies are defined as a set of skills, knowledge, attitudes and values that are interdependent and necessary for the execution of actions, aiming at an efficient performance in the exercise of professional activity^(^ 5 ^-^ 6 ^)^.

In the context of the health area, more specifically in the training of nurses, skills extrapolate psychomotor skills, expanding to development based on cognitive, emotional and social skills that converge for decision making and problem solving^(^ 5 ^-^ 7 ^)^. In Brazil, nursing graduates are expected to have a generalist, humanistic, critical and reflexive education, with a view to acting, in a resolutive way, on health indicators, from integral and transdisciplinary care^(^ 7 ^-^ 9 ^)^.

In view of the above, it is understood that the profile of the nurse expressed in the National Curricular Guidelines of the Undergraduate Nursing Course (NCD/ NUR) is of a professional with competencies, skills, attitudes and values to make assertive decisions in the different needs / realities and levels of attention^(^ 7 ^-^ 8 ^,^ 10 ^-^ 11 ^)^. Thus, training that promotes professional autonomy, inter and trans-disciplinarity, capacity for self-learning, approximation of health services and focus on ethical and integral care becomes decisive in the qualified preparation of future nurses^(^ 9 ^-^ 10 ^)^.

In this sense, a study conducted with 505 graduates assessed the skills and abilities developed during graduation from aspects expressed in the NCD/ ENF. For these graduates, it was evidenced the need for greater stimulation of autonomy, exchange of experiences and early insertion in health services^(^ 5 ^)^.

Other studies^(^ 10 ^-^ 12 ^)^ consider promising the curricular proposal that works the competences from the early insertion of the student in the scenarios of practices. This process facilitates the integration of teaching-service-community by mediating the student’s contact with the unique and plural challenges of the profession. These proposals include the National Program for the Reorientation of Vocational Training in Health (*Pró-Saúde*) and the Health Work Education Program (*PET-Saúde*), which propose, in their bases, the inseparability between teaching, research, extension and service^(^ 11 ^-^ 14 ^)^.

However, despite the fact that NCDs/NUR act as the guiding axis in the construction of the egress profile, studies^(^ 5 ^,^ 13 ^-^ 15 ^)^point out the importance of conducting research that seeks to understand the nurses’ training process from the point of view of the egress, thus enabling the improvement and restructuring of curricula in line with local and national demands.

In this logic, the perspective of the graduates can operate as an indicator of the formative process and of the professional insertion, allowing comparisons, adjustments and curricular changes when pertinent. This premise justified the accomplishment of this investigation, whose objective was to evaluate the profile of the graduates of Nursing of a public college from the perception of competences developed during the graduation and of the process of professional insertion.

## Method

This is an exploratory and descriptive study, with a quantitative approach, carried out with registered nurses from a School of Public Nursing in the State of Minas Gerais. In this institution, currently, the baccalaureate is developed in ten semesters and in full time. 40 students are admitted per semester, through the Joint Selective Entrance Program (Pism) and the Unified Selection System (SISU) of the Ministry of Education.

Data collection was carried out between August 2017 and April 2018. The study population consisted of 470 graduate graduates between 2005 and 2017. The inclusion criteria were: to have e-mail registered in the coordination of undergraduate course between the years 2005 and 2017. The year 2005 was established as the minimum inclusion threshold, since it was considered that the first group was formed after the promulgation of the NCD/NUR of 2001. Students were excluded who did not complete and / or dropped out of the course and who did not respond the request to complete the questionnaire even after five attempts.

A sample calculation was established for the finite population, with 95% confidence and error of 5%, considering an estimate of the proportion equal to 50%. A sample size of 212 was obtained to meet the statistical validity requirement. In this sense, a sample of 216 graduates.

The data was collected by applying a validated instrument^(^ 16 ^)^, adapted for online research using the Google Docs Forms tool with semi-open, multiple-choice and Likert-type scales.

The instrument^(^ 16 ^)^ is composed of three dimensions: sociodemographic characteristics, with 13 closed and semi-closed questions; characterization of the insertion and the professional trajectory, with nine questions of multiple choice and evaluation of the process of professional formation, with eight questions constructed in Likert type scale, one to five, in which one means “I totally agree and five”, “Disagree fully”.

The data obtained through the online questionnaire were tabulated in an Excel - Microsoft Office 2010 worksheet and then transferred to the analysis in the Statistical Package for Social Science (SPSS) software, version 21.

The descriptive and exploratory data analysis was performed using absolute and relative frequencies, mean and standard deviation, and the correlation between qualitative variables by the chi-square test.

The study complied with all ethical precepts, with approval of the project by the Research Ethics Committee under CAAE No. 66674917.5.0000.5147 and opinion no. 2,253,442.

## Results

Of the total of 470 eligible graduates, two (0.4%) were excluded because they did not have electronic mail registered at the university, 89 (18.9%) dropped out / did not complete the course and 163 (34.7%) did not respond to the questionnaire. Thus, 216 graduates (46%) participated in this study, of which 56% completed the course between 2011 and 2016. Regarding the sociodemographic data, the majority were female, with a mean of 29.62 years (SD = 8.2) and with a self-declared white color. As for the place of residence, the majority were residing in the State of Minas Gerais and 23.1% in other Brazilian states, especially Rio de Janeiro (Table 1).

**Table 1:** Sociodemographic characteristics of graduates of the Undergraduate Nursing Course (n = 216), Minas Gerais, Brazil (2017-2018)

Characteristic	N	%
**Sex**		
Female	191	88
Male	25	12
**Colour**		
White	143	66
Brown	57	26
Black	13	6
Yellow	1	0.5
Not declared	2	1
**Place of residence**		
Juiz de Fora	122	56
Municipalities of Zona da Mata mineira	21	9.7
Capital and metropolitan region	7	3.2
Interior of the State	15	6.9
Other States of Brazil	50	23.1
Another country	1	0.6
**FU**		
Minas Gerais	165	76.3
Rio de Janeiro	36	16.6
São Paulo	7	3.2
Distrito Federal	3	1.4
Bahia	2	0.9
Mato Grosso do Sul	1	0.4
Rio Grande do Sul	1	0.4
Other (Iowa/ EUA)	1	0.4
**Year of graduation**		
Until 2005	4	2%
From 2006 to 2010	91	42%
From 2011 to 2015	75	35%
From 2016 onward	46	21%

Regarding the academic activities carried out during the graduation period, 188 graduates (86%) participated in projects or programs linked to extension and research and 132 (61%) reported having acted as monitors.

Regarding the academic trajectory after graduation, the majority of the graduates (44.5%) were studying or had a Stricto sensu postgraduate course, followed by 43% who were attending or had a specialization in Lato sensu.

Regarding the professional insertion after graduation, 48% of the graduates started the activities in up to six months and the majority (67%) got the first job within a year after graduation. As for the employment situation, it was verified that the majority (65%) had a bond in which they are active as a nurse. Of these, 14% worked in a single institution. It should be noted that 41 graduates (19%) reported being unemployed at the time of the research and 35 (16%) reported having a work bond outside Nursing.

There was a significant association between the year of graduation and the fact of having or not employment. For those who completed college by 2010, statistical significance was found (p <0.001), with more people employed in the graduation period from 2007 to 2010 than from 2011 to 2017.

Among the areas of professional activity, a significant proportion (56%) worked in care, followed by education (30%). Regarding the characterization of the professional trajectory, it was verified that 77 (36%) entered the profession by public competition, followed by 42 (19%) who did it through a selection process. Regarding the nature of the institution in which it worked at the time of data collection, the majority (47%) worked in the public network and 17 (8%) in private institutions. As for the monthly income, 50% had a salary range between two and seven salaries, with an average of 4.5 minimum salaries. Regarding the weekly workload, the highest frequency (38%) of responses was between 37 and 44 hours per week and the lowest (3%), with a weekly workload of more than 60 hours.

The responses to the competences recommended for an integral formation of the nurse professional, with emphasis on the NCD/ NUR and UHS principles, had their results summarized, according to Table 2.

**Table 2:** Distribution of the answers regarding the competences perceived for the exercise of activities inherent to the profession (n = 216), Minas Gerais, Brazil (2017-2018)

Activities inherent to the profession	TD*	D^†^	NDA^‡^	A^§^	TA^‖^
Promotion / prevention / protection / rehabilitation at various levels	4%	8%	7%	51%	30%
Actions to different needs	4%	9%	12%	46%	30%
Men’s health	8%	24%	37%	23%	8%
Women’s health	4%	6%	6%	43%	40%
Newborn’s health	6%	17%	16%	47%	14%
Child and adolescent health	3%	12%	19%	49%	17%
Adult Health	6%	8%	12%	48%	27%
Health of the Elderly	5%	13%	23%	47%	13%
Apply Nursing Process	6%	10%	22%	40%	22%
Integrality of assistance	3%	11%	10%	44%	31%
Make decisions	6%	8%	15%	46%	25%
Being a processing agent	4%	6%	14%	46%	30%
Ethical and legal principles	6%	2%	3%	31%	58%
Manage with ethics and bioethics	6%	2%	6%	36%	50%
Keep information confidential	6%	1%	3%	29%	60%
Comply with Council rules	5%	5%	8%	35%	47%
Plan / participate in surveys	5%	16%	21%	39%	19%
Evidence-based practice	5%	14%	25%	40%	16%
Political / planning activities	5%	10%	14%	44%	28%
Coordinate the caring process	5%	5%	10%	50%	29%
Training of human resources	3%	7%	17%	46%	27%
Permanent education program	3%	5%	17%	49%	26%
Health Education / Promotion Program	4%	5%	16%	49%	27%
Coordinate nursing team	5%	7%	13%	41%	34%
Manage conflicts	7%	13%	23%	36%	22%
Manage the work process	2%	14%	16%	47%	20%
Cooperate with the health team	4%	7%	10%	45%	33%
Cost-effectiveness / benefit analysis	9%	19%	24%	30%	19%

* Strongly disagree;

† Disagree;

‡ Neither disagree nor agree;

§ Agree;

‖ I totally agree

The first nine items evaluated in the scale are related to the competence for the private activities of the nurse, in each field. In this context, considering “I agree” and “totally agree”, there was a prevalence (81%) of those who evaluated that graduation prepared them to carry out promotion, prevention, protection and rehabilitation actions, from the perspective of integral care, followed by 83% who felt able to work in comprehensive care programs for women’s health. However, with regard to the competence to act in the programs of integral assistance to the Health of Man, 31% of the graduates recognized to be able to. Regarding the competence to attend the patient comprehensively, items 10 to 12, the predominance of responses that indicate abilities to promote healthy lifestyles and to reconcile the needs of the users.

When evaluating the competences about ethical issues in Nursing practice, items 13 to 16, it is noticed that, among all blocks of questions, this was the one that presented the highest frequency of positive answers. For the graduates analyzed, 58% and 60%, respectively, responded fully agreeing that during graduation they learned to respect the ethical, legal and humanistic principles of the profession and to ensure the privacy of the user attended, ensuring the complete confidentiality of information acquired during the service.

It was also evaluated the technical and scientific competence to exercise Nursing. In this block of items, 17 and 18, there was a positive trend, with 58% of the graduates believing that they were trained during graduation to plan and produce scientific knowledge through research in the area of Nursing. Concerning the recognition of the social role of the nurse in political activities and health planning, coordinating all phases of the Nursing care process, items 19 and 20, most of the graduates felt empowered.

With regard to health education, items 21 to 23, most graduates evaluated having been prepared to plan, implement and participate in continuing education programs in the service. Similarly, for the block of questions about teamwork and conflict management, items 24 and 25, there was consonance for positive responses. However, the competence for coordinating Nursing team activities received more positive responses than the items that evaluated the competence to administer conflicts of the Nursing and multi-professional team.

In the block that evaluates competence for health management, items 26 to 28, the cooperation with the health management team was highlighted in relation to the items that evaluated aspects of work process management and cost-effectiveness, cost- benefit and cost-utility of health products and procedures.

It should be noted that, following the application of the questionnaire, the Cronbach Alpha Coefficient was tested, and high reliability ( > 0.8) was verified for the instrument used in data collection.

## Discussion

From the sociodemographic data, it was observed that the participants of this study were predominantly young adults, whites and females. Corroborating this result, further research^(^ 5 ^,^ 17 ^-^ 18 ^)^ carried out with Nursing graduates have indicated the predominance of women and the average age group below 32 years.

Globally, international studies^(^ 17 ^-^ 19 ^)^ highlight the growing insertion of women under the age of 30 in the world of work, with emphasis on Nursing in the health area. However, regarding employability, Nursing has experienced a demand for jobs below the market’s offer in the South and Southeast regions of the country^(^ 20 ^)^.

Similar to other studies^(^ 5 ^,^ 18 ^,^ 20 ^)^, the majority of graduates remained in the region in which they graduated. In Brazil, there is a significant number of nurses in the Southeast region, with the states of São Paulo (24.6%), Rio de Janeiro (11.1%) and Minas Gerais (10.4%). On the other hand, it is evident the shortage of professionals in poorer regions such as North and Northeast, provoking inequalities of healthcare coverage in these localities^(^ 21 ^)^.

In relation to the extra-class activities developed by the graduates during graduation, there was mention of extension and monitoring, followed by the research. These activities, with emphasis on the university extension, enable a critical, creative and resolute training, geared towards the attendance of the most varied social demands of health linked to the world of work. It should be noted that in Nursing, the insertion of academics into groups and scientific productions is still incipient and indicates the need to work on the inseparability between teaching, extension and research^(^ 5 ^,^ 22 ^-^ 24 ^)^.

With regard to specialization, it was evidenced that part of the graduates sought qualification in postgraduate courses Stricto sensu and Lato sensu. However, a congener study, conducted in the State of São Paulo, found a greater insertion in Lato sensu postgraduate courses (63.9%)^(^ 5 ^)^.

Regarding the insertion in the world of work, it was verified that the majority began to exercise the profession within 12 months after graduation, with prevalence of the assistance area, receiving, on average, 4.5 minimum wages, for up to 44 weekly hours of work.

It was found, corroborating the data, that a study carried out with 172 graduates from a public college found that 52.9% of the nurses entered the profession within six months after graduation^(^ 5 ^)^. However, another study showed that, although most of the participants were employed, there were reports of difficulties in entering the labor market^(^ 25 ^)^. In this sense, it is pertinent to highlight the natural difficulties to obtain the first job, mainly due to the lack of previous experience, the saturation of the labor market in regions such as the Southeast or even the incompatibility with the profile demanded by the employer. The realization of extracurricular internships and specialization in the modality of residence has been a good option of in-service training that mediates the transition from university to the world of work^(^ 24 ^)^.

With regard to the competences developed during graduation, there were predominant answers that go to what establishes the NCD / NUR and the formation for the UHS. In this sense, the participants expressed that graduation enabled them to take decisions and intervene in health problems from a comprehensive and effective care at different levels of health care.

Consonant with these results, a search ^(^ 5 ^)^ described that 62% of the graduates agreed that graduation enabled the development of competencies related to promotion, prevention, protection and rehabilitation at the various levels of attention, from the perspective of integrality. However, studies^(^ 5 ^,^ 25 ^)^ carried out with nurses revealed that, upon reaching the labor market, they identified limited preparation, difficulty to make decisions and to confront the specific healthcare reality of hospitals, and it was necessary to search for specialization courses.

It should be noted that, in this investigation, few agreed that, upon graduation, they were able to participate in comprehensive health care programs. Unlike programs that have historically achieved advances, such as maternal and child health, the man’s health program is a recent policy, necessitating, therefore, strategies that will qualify future nurses to intervene in the health of this population. In this sense, studies^(^ 26 ^-^ 27 ^)^ have indicated low adherence of men to health services, which raises, in the undergraduate curricular strategies transversal on the health of the man and programs of permanent education in service.

Regarding the competence to act on the ethical issues, it was observed that, like other investigations, the graduates felt able to act before legal and humanistic aspects of the profession based on the principles of bioethics^(^ 5 ^,^ 25 ^)^. A Finnish study ^(^ 28 ^)^ highlighted the perceived ability of newly graduated nurses to decision-making guided by ethical values (86.8%). Another study^(^ 29 ^)^, conducted with newly trained nurses, found a strong correlation between basic competences to the exercise of Nursing and ethical and bioethical aspects.

In this study, the majority of graduates evaluated positively the course regarding the acquired competences for the technical-scientific performance of the profession in political activities, planning, health education and permanent education. It should be noted that, although the competencies for the management activities were evaluated positively, when compared with the other skills, this group of answers obtained the lowest score, with emphasis on conflict management and cost- effectiveness. In some way, this is related to the specifics of each organization / institution, in addition to a formation that is not linked to real situations of work practice. Studies^(^ 25 ^,^ 29 ^-^ 31 ^)^ point out that, in addition to the challenges of starting the career, the young nurse as a service manager / nursing team often faces dilemmas such as limited human and material resources, conflicting interpersonal relationships, rigid organizational management and varied care demands.

In this sense, it is necessary to emphasize on the need of strategies that approach the academic experiences of nurses’ daily work. In addition, permanent education, mediated by educational institutions and/or services, is an important tool for the production of skills, professional values and qualification of care^(^ 31 ^)^.

Finally, it is necessary to reflect on the Nurse of the future nursing core competencies^(^ 32 ^)^, *which strategically lists ten essential competences for the training and practice of the nurse of the future, namely: patient-centered care; professionalism; leadership; systems-based practice; ability in computer science and technology; Communication; collaborative teamwork; safety; quality improvement and evidence-based practice* ^(^ 32 ^-^ 33 ^)^ *. These competences (re) outline the contemporary profile of the egress, socially, ethically and politically committed to the profession and the health of the population* ^(^ 33 ^)^.

Regarding the limitations of the study, we indicate the accomplishment of research in a single educational institution and the absence of a qualitative evaluation that allowed to verify the meanings and meanings of the developed competences and the process of professional insertion.

In addition, some nurses who participated in the study had up to 12 years of training and certainly more professional experience compared to the newly graduated. This variable may have generated heterogeneity in the information provided and / or difficulty to evaluate the competences, considering that the changes are constant in the training process.

However, the research results provide contributions to elucidate the nurses’ training process, their potentialities and limitations, their insertion in the labor market, as well as to strengthen reflections on the proposal of the new NCD/NUR that are under discussion in the country.

## Conclusion

The research made it possible to delineate the profile of the Nursing graduates of a public college from the perception of the competences developed during graduation and the process of professional insertion. Most of the participants are women, are in the region where they graduated and got the first job within one year after graduation.

During graduation, the most frequent extraclass activity was university extension. For graduate students, graduation enabled the development of competencies for health education, promotion, prevention, protection and rehabilitation at the various levels of attention from the perspective of bioethics. They express that they have been prepared to act as social agents in the political and health planning and nursing activities. However, compared to other competencies, there was a lower score for the ability to work in programs of integral assistance to human health, conflict management and cost-effectiveness analysis.
